# Targeted mapping and utilization of the perihepatic surface for therapeutic beta cell replacement and retrieval in diabetic non-human primates

**DOI:** 10.3389/frtra.2024.1352777

**Published:** 2024-01-26

**Authors:** David J. Leishman, Scott H. Oppler, Laura L. Hocum Stone, Timothy D. O’Brien, Sabarinathan Ramachandran, Bradley J. Willenberg, Andrew B. Adams, Bernhard J. Hering, Melanie L. Graham

**Affiliations:** ^1^ Preclinical Research Center, Department of Surgery, University of Minnesota, Minneapolis, MN, United States; ^2^ Department of Veterinary Population Medicine, University of Minnesota, St. Paul, MN, United States; ^3^ Department of Internal Medicine, University of Central Florida College of Medicine, Orlando, FL, United States; ^4^ Division of Transplantation, Department of Surgery, University of Minnesota, Minneapolis, MN, United States; ^5^ Schulze Diabetes Institute, Department of Surgery, University of Minnesota, Minneapolis, MN, United States

**Keywords:** islet transplantation, transplantation site, perihepatic surface, biomaterials, engraftment, type 1 diabetes mellitus, beta cell replacement, hydrogels

## Abstract

**Introduction:**

Successful diabetes reversal using pancreatic islet transplantation by various groups illustrates the significant achievements made in cell-based diabetes therapy. While clinically, intraportal islet delivery is almost exclusively used, it is not without obstacles, including instant blood-mediated inflammatory reaction (IBMIR), relative hypoxia, and loss of function over time, therefore hindering long-term success. Here we demonstrate the perihepatic surface of non-human primates (NHPs) as a potential islet delivery site maximizing favorable characteristics, including proximity to a dense vascular network for adequate oxygenation while avoiding IBMIR exposure, maintenance of portal insulin delivery, and relative ease of accessibility through minimally invasive surgery or percutaneous means. In addition, we demonstrate a targeted mapping technique of the perihepatic surface, allowing for the testing of multiple experimental conditions, including a semi-synthetic hydrogel as a possible three-dimensional framework to improve islet viability.

**Methods:**

Perihepatic allo-islet cell transplants were performed in immunosuppressed cynomolgus macaques using a targeted mapping technique to test multiple conditions for biocompatibility. Transplant conditions included islets or carriers (including hydrogel, autologous plasma, and media) alone or in various combinations. Necropsy was performed at day 30, and histopathology was performed to assess biocompatibility, immune response, and islet viability. Subsequently, single-injection perihepatic allo-islet transplant was performed in immunosuppressed diabetic cynomolgus macaques. Metabolic assessments were measured frequently (i.e., blood glucose, insulin, C-peptide) until final graft retrieval for histopathology.

**Results:**

Targeted mapping biocompatibility studies demonstrated mild inflammatory changes with islet-plasma constructs; however, significant inflammatory cell infiltration and fibrosis were seen surrounding sites with the hydrogel carrier affecting islet viability. In diabetic NHPs, perihepatic islet transplant using an autologous plasma carrier demonstrated prolonged function up to 6 months with improvements in blood glucose, exogenous insulin requirements, and HbA1c. Histopathology of these islets was associated with mild peri-islet mononuclear cell infiltration without evidence of rejection.

**Discussion:**

The perihepatic surface serves as a viable site for islet cell transplantation demonstrating sustained islet function through 6 months. The targeted mapping approach allows for the testing of multiple conditions simultaneously to evaluate immune response to biomaterials at this site. Compared to traditional intraportal injection, the perihepatic site is a minimally invasive approach that allows the possibility for graft recovery and avoids IBMIR.

## 1 Introduction

In the United States, over 11% of the population has been diagnosed with diabetes (1), with the incidence and prevalence of the disease continuing to grow on a national and global level (2). While only representing approximately 10% of diabetes diagnoses, type 1 diabetes mellitus (T1D) is typically diagnosed earlier than type 2 diabetes mellitus (T2D), often at approximately 4–5 years of age or in the teenage years (3, 4); this leads to a longer duration living with the disease and a greater risk for the long-term complications associated with diabetes (5, 6). The standard of care for T1D includes frequent blood glucose monitoring along with exogenous insulin administration, a non-physiologic treatment often associated with a greater burden of disease and reduced quality of life for patients (7–10).

For some patients with T1D, pancreatic islet transplantation is an option to replace lost β-cells and recapitulate endogenous insulin secretion (11, 12). Allogeneic islet cell transplantation has been shown to result in near-normoglycemia (13), decreased hypoglycemic episodes in brittle diabetics (14–16), and improvement or slowing of the micro- and macrovascular complications of T1D (17–22). Despite this success, the widespread adoption of this cell-based therapy has been hindered by an insufficient supply of donor organs, deterioration of graft function over time, and side effects from life-long immunosuppression (10, 23–26). These obstacles are in part due to the injection of islets into the portal vein, the site of choice since the beginning of clinical islet transplantation (11, 27).

Though used as the clinical transplantation site, the portal vein is far from ideal. Islet cells are particularly susceptible to hypoxemia and the portal vein oxygen tension is well below that of the pancreas (28–30). Furthermore, intravascular injection subjects the transplanted islets to instant blood-mediated inflammatory reaction (IBMIR), resulting in inflammation, further hypoxemia, exposure to inflammatory cells and blood components, and the complement cascade (11, 12, 30, 31). It has been estimated that between 50% and 70% of islet grafts are immediately destroyed as a result of IBMIR (32), which explains, in part, the need for significant amounts of islets for each recipient. When introduced intraportally, the islets embolize the liver, further amplifying the hypoxia while also contributing to liver steatosis, another factor implicated in graft loss (33, 34). Portal vein implantation also exposes islets to higher immunosuppressive drug concentrations than in the periphery (35, 36), reaching levels known to cause destruction of islets (37) or inhibit angiogenesis and healing (38), which is of great consequence during islet engraftment. While all these features impact long-term outcomes, thus far, no other site has demonstrated consistent successful engraftment and metabolic benefit in large animals or the clinic. As a result, specific attention has been turned to possible alternative sites to the portal vein for pancreatic islet transplantation.

An optimal site would not only offer the efficient engraftment of islets but also capture the physiologic secretion to maximize metabolic benefit without increasing the number of islets needed to reverse diabetes. The site should have a rich vascular supply to boost the oxygen tension for the islets, create an ideal microenvironment to prevent early loss to promote engraftment, protect from rejection and IBMIR, and recapitulate portal venous drainage to avoid systemic hyperinsulinemia (39, 40). Ideally, the site would be relatively easy to access to minimize significant surgery and allow for biopsy or access for functional assessment (30, 41, 42). To this end, others have investigated various means to address some of these issues including different cell encapsulation techniques using semi-permeable barriers to immunoisolate cells or other anatomic sites, including the subcapsular kidney, gonadal fat pad, peritoneum, gastrointestinal wall, spleen, pancreas, and intramuscular and subcutaneous space (10, 30, 39, 43, 44). Most of these have been studied in experimental rodent models and have never been tested in large animal studies related to a lack of clinical relevance or scalability.

Given this, our experimental goal was to utilize a site distinct from the traditional intraportal site and functionalize it to support transplanted islet cells. The perihepatic (PH) liver surface was chosen for multiple reasons: (1) the liver is a highly perfused and well-vascularized organ receiving approximately 25% of cardiac output (45) with the perihepatic surface creating a prevascularized bed; (2) islets will have close proximity to a dense vascular network but will be protected against IBMIR; (3) presumed engraftment in the PH will allow for physiologic portal drainage of insulin via the sinusoids and reinnervation for potential normal pulsatile secretion; (4) the PH surface is easily accessible for transplantation, biopsy, or graft retrieval via minimally invasive surgery or percutaneous ultrasound guidance with little detriment to liver functionality (46, 47); and (5) the large surface area of the human liver (approximately 1,000 cm^2^ (48)) would provide plenty of area for a superficial graft.

We also demonstrate a unique method of targeted mapping of the PH surface to quickly and efficiently test various transplantation conditions to determine the most suitable environment for the islet grafts. In these experiments, we used small volumes of islets and attempted to further functionalize the PH surface with carrier constructs, including a three-dimensional capillary alginate hydrogel. Carriers can serve as mechanical support and provide spatial distribution to the islets. Hydrogel scaffolds can support islets that are particularly vulnerable after the isolation and purification process, having damaged or lost extracellular matrix (ECM) or basement membranes (49). These hydrogels are similar to natural tissue ECM and can facilitate rapid revascularization (50). Moreover, hydrogel-islet constructs that are injectable, allow for extremely precise injection and localization of grafts. By utilizing the PH surface, a prevascularized area with relative ease of access and potential for physiologic insulin secretion, we aim to improve islet cell engraftment to establish long-term graft function while providing a means for monitoring and biopsy.

## 2 Materials and methods

### 2.1 Animal subjects

All animal procedures were approved by the University of Minnesota Institutional Animal Care and Use Committee, conducted in compliance with the Animal Welfare Act, adhered to principles stated in the NIH Guide for Care and Use of Laboratory Animals (51), and were performed and reported in compliance with the ARRIVE guidelines. All animals were purpose-bred and purchased from institutionally approved commercial vendors. Animals used in this study were assigned to study group/experimental conditions based on appropriateness for study; due to the study's purpose and exploratory nature, no animals were assigned to a conventionally defined control group. Due to clinical care requirements, experimenters could not be blinded to an animal's experimental condition for certain aspects of the experiment, including metabolic characterization. Blinding occurred during data analysis when feasible.

#### 2.1.1 Non-human primates

A total of five Mauritian-origin cynomolgus macaques (*Macaca fascicularis*) (four female, one male) were enrolled for testing. All enrolled animals were healthy and confirmed to be tuberculosis (TB) negative and viral negative (macaque herpes B virus, simian retrovirus D, simian immunodeficiency virus, and simian T-cell leukemia virus-1). The mean age of the animals was 6.1 ± 2.0 years and their mean weight was 5.1 ± 1.9 kg. For this exploratory study, each individual animal was used to model a combination of conditions of interest, enrolling one of the commonly used species of macaques used in transplantation modeling. These studies were not designed to achieve statistical significance or detect rare adverse events. Animals are presented individually for clarity and, where appropriate, grouping by similar experimental condition has been performed to evaluate trends and define expected variability for future modeling.

To realize the need for frequent blood draws while avoiding confounding effects from restraint, sedation, and pain, all animals were implanted with single-incision, peripherally inserted vascular access ports (VAPs) as previously described (52). All animals were trained to cooperate with examination, blood collection, and general husbandry activities as part of the behavioral management program (53, 54).

Animals were fed a standardized diet of either 2055C Certified Teklad Global 25% Protein Primate Diet or 7195 Teklad High Fiber Primate Diet (Envigo, Madison, WI, USA). A standardized enrichment program was used for the duration of the study, including fresh fruits and vegetables, grains, beans, and nuts, as well as a children's multivitamin.

Animal behavior and clinical status were evaluated at least twice daily. Scheduled physical examinations per protocol and semi-annual comprehensive veterinary examinations were performed on all animals. Animals were continuously housed in same-sex pairs, except in rare cases of demonstrated social incompatibility, in which singly housed animals remained in close proximity with social conspecifics maintaining visual, auditory, and olfactory contact at all times until re-pairing. An environmental enrichment program including social play, toys, music, and regularly scheduled access to a large exercise and swimming area was provided to encourage sensory engagement, enhance foraging behavior and novelty seeking, promote mental stimulation, increase exploration, play, and activity levels, and strengthen social behaviors, increasing the proportion of time animals spent on species-typical behaviors. All animals enrolled in this study were offered equal access and time for exercise and identical enrichment activities.

### 2.2 Diabetes induction

Diabetes was induced in three animals using pharmaceutical grade STZ (streptozotocin, Zanosar; Sicor Pharmaceutics, Irvine, CA, USA) using methods previously described by this laboratory (55, 56). After verifying appropriate hydration, a single dose of 100 mg/kg STZ was infused IV. Diabetes was confirmed by persistent hyperglycemia (>300 mg/dl on at least two consecutive readings), the need for exogenous insulin to maintain target blood glucose levels, and the absence of a C-peptide response to metabolic challenge. Non-human primates (NHPs) with diabetes were treated using glargine and lispro in combination on a sliding scale to target preprandial blood glucose levels between 50 and 200 mg/dl.

### 2.3 Hydrogels

**Figure 1:**
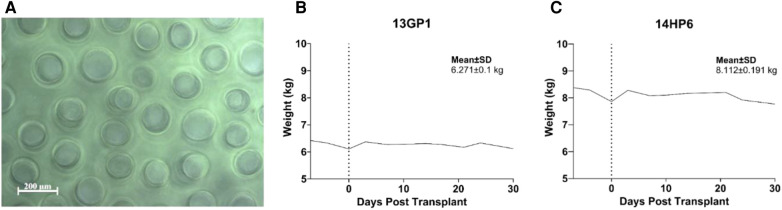
Biomaterial targeted mapping evaluation. (**A**) Phase-contrast image of parallel microchannels in capillary alginate hydrogel used in targeted mapping. (**B** and **C**) Biocompatibility and general safety of approach was assessed in non-diabetic NHPs. Weight trend with overall mean and standard deviation is presented.

Capillary alginate gel (Capgel™) is a self-assembled hydrogel comprising alginate and optionally other biopolymers, such as gelatin (57–64), with unique microstructures of packed parallel capillary channels running the length of the material (Figure 1A). Capgel™ was synthesized as has been extensively described in previous publications (57–64). Specifically, the formulation of all parent gel solutions was 2% w/v alginate (Pronova®, NovaMatrix®; Sandvika, Norway) and 2.6% w/v gelatin (Sigma-Aldrich, St Louis, MO, USA), and parent gels were grown with 0.5 M copper (II) sulfate pentahydrate (CuSO4 5H2O; Acros Organics, Fisher Scientific, Thermo Fisher Scientific Inc., Waltham, MA, USA). Once self-assembly was completed, the parent gels were rinsed extensively, sectioned, crosslinked in the cold using carbodiimide chemistry (Sigma-Aldrich, St Louis, MO, USA), processed, sterilized via autoclave, and the final Capgel™ product stored at 4°C until used.

### 2.4 Immunosuppression protocol

Antagonistic anti-CD40 mAb 2C10R4, provided by the NIH Nonhuman Primate Reagent Resource, was given IV at 50 mg kg on days −7, −1, 7, and 14, and then every 14 days. Rapamycin was given PO from day −7 through study termination; the target trough level was 5–12 ng ml. NHPs in the targeting mapping cohort were terminated at day +30, with the last dose of anti-CD40 on day +14 and rapamycin on day +29. Concomitant anti-inflammatory therapy consisted of αIL-6R (tocilizumab, Actemra®) at 10 mg/kg IV on days −7, 0, 7, 14, and 21, and sTNFR (etanercept, Enbrel®) at 1 mg/kg IV on days −7 and 0 and 0.5 mg/kg SC on days 3, 7, 10, 14, and 21.

### 2.5 Islet isolation and quality control

Adult cynomolgus macaque islets were isolated and cultured as previously described and evaluated for conventional quality control (purity, sterility, and viability assessed by oxygen consumption rate normalized for DNA) (65).

### 2.6 Anesthesia and analgesia

For surgical procedures and euthanasia, anesthesia was induced with 10–12 mg/kg ketamine IM with or without 0.1 mg/kg midazolam IM and 0.5%–3% isoflurane inhaled for maintenance anesthesia. Post-operative analgesia was administered for at least 72 h with 0.01–0.03 mg/kg buprenorphine IM BID and 1.0 mg/kg ketoprofen IM daily for pain management.

### 2.7 Islet transplantation and biopsy

#### 2.7.1 Islet-hydrogel mapping surgery

After the induction of anesthesia, NHPs were intubated and positioned supine. The intended incision sites were clipped of hair and the sites were widely prepped with chlorhexidine gluconate/isopropyl alcohol solution and draped with sterile towels. The incision sites were infiltrated with 1% lidocaine (1:5 dilution). A 6 cm midline incision was made caudal to the xiphoid process. A gentle blunt dissection was used to expose the linea alba, which was then incised, and the peritoneum entered. The liver was immediately visualized, and a padded Babcock clamp was placed on the edge of the left lateral liver lobe. The lobe was gently externalized and then held by hand to expose the capsule for injection. Various islet constructs with or without carriers were injected into the left lateral lobe of the liver (injection volume per site: 100–250 µl) using a 25 g needle just under the capsule. A notable wheal was formed under the capsule for each injection. Each wheal was made equidistant from one another, and gentle pressure was held after each injection to ensure no leakage of islet product. For a given NHP, the islet product was equally divided across each injection site. There was minimal to no bleeding visualized at each injection site. The Babcock clamp was then removed, and the liver gently replaced into the abdomen. The incision was closed in five layers using 5–0 absorbable monofilament suture and sealed with topical skin adhesive.

#### 2.7.2 Islet transplantation in NHPs with diabetes

After the induction of anesthesia, NHPs were intubated and positioned supine. The intended incision sites were clipped of hair and the sites were widely prepped with chlorhexidine gluconate/isopropyl alcohol solution and draped with sterile towels. The incision sites were infiltrated with 1% lidocaine (1:5 dilution). A 2–3 cm midline incision was made caudal to the xiphoid process. The liver was immediately visualized, and a padded Babcock clamp was placed on the edge of the left lateral liver lobe. The lobe was gently externalized and then held by hand to expose the capsule for injection. Using a 22 g angiocatheter, saline was used to hydrodissect the liver capsule from the parenchyma and was then subsequently drawn back into the syringe. Islets in autologous plasma were injected using the same angiocatheter just under the capsule where it had been hydrodissected (injected volume 250–900 µl). A notable wheal was formed under the capsule. Gentle pressure was held after the injection to ensure no leakage of islet product and skin adhesive was used, if needed, to seal the puncture site. There was minimal to no bleeding visualized at each injection site. The Babcock clamp was then removed, and the liver gently replaced into the abdomen. The incision was closed in five layers using 5–0 absorbable monofilament suture and sealed with topical skin adhesive.

### 2.8 Euthanasia

Anesthesia was induced as described in Section 2.6 and the animals were euthanized using a barbiturate overdose consisting of 87 mg/kg pentobarbital +11 mg/kg phenytoin (Beuthanasia) IV.

### 2.9 Laboratory testing

For complete blood counts, venous blood samples were collected into EDTA-treated microtainers and analyzed using the Advia 2120 hematology analyzer (Siemens Healthineers USA, Malvern, PA, USA). For chemistry panels, venous blood samples were collected into serum separator tubes and centrifuged to obtain serum. Chemistry panels were analyzed using an AU480 chemistry analyzer (Beckman Coulter, Brea, CA, USA).

### 2.10 Graft assessment

#### 2.10.1 Laboratory testing

Point-of-care glucose measurements were made using a standard glucometer (Nova Biomedical, Waltham, MA, USA). HgbA1c was measured from whole blood using a point-of-care DCA Vantage Analyzer (Siemens Healthineers USA, Malvern, PA, USA). For C-peptide assays, venous blood was collected into serum separator tubes treated with bovine lung aprotinin (Millipore-Sigma, Darmstadt, Germany) at a ratio of a minimum of 500 kU to 1 ml of sample. C-peptide was measured via radioimmunoassay (Millipore-Sigma, Darmstadt, Germany) using the Genesys Genii instrument (Laboratory Technologies, Elburn, IL, USA).

#### 2.10.2 Glucose tolerance testing

Briefly, animals were fasted overnight. For intravenous glucose tolerance testing (IVGTT), ≤25% dextrose (0.5 g/kg) was injected and the VAP was immediately flushed with normal saline at 10× port and catheter volume to assure there was no residual dextrose contamination in subsequent samples; blood was collected at multiple timepoints (3× baseline, 1, 3, 5, 7, 10, 15, 20, 25, 30, and 60 min) in awake, cooperating animals. Additional glucose measurements at 15, 20, 25, 30, and 60 min were obtained via heel stick.

The glucose disappearance rate (K_glucose_) was calculated as the slope of the decline of the log-transformed blood glucose between 10 and 30 min.

#### 2.10.3 Histological processing

Islet cells were fixed in 10% formalin, paraffin-embedded, and processed for routine histology. Immunohistochemistry was performed on retrieved islet graft sites taken from the PH surface. Sections of tissue with a thickness of 4 µm were cut and slides were loaded onto the Biocare Intellipath IHC staining instrument (Biocare Medical, Pacheco, CA, USA). Slides were deparaffinized through xylene and rehydrated through graded alcohol to water. If needed, heat retrieval was performed. Endogenous peroxide was quenched with 3% hydrogen peroxide followed by a protein serum block. Antibodies were applied followed by detection, each for 30 min at room temperature. Slides were developed with DAB and counterstained with Mayer’s hematoxylin, insulin, CD3, CD20, IBA-1, CD31, and β3 tubulin IHC staining.

After staining, biopsies were imaged using a Nikon Eclipse-800M bright-field/fluorescence/dark-field microscope equipped with a Nikon DXM1200 high-resolution digital camera and NIS Elements-D 5.02.00 Imaging software.

#### 2.10.4 Histological assessment

All islet graft sites from the PH surface were reviewed by a board-certified veterinary pathologist and scored to assess the degree of insulin immunoreactivity, infiltration of the graft constructs by immune and inflammatory cells, vascularization, and innervation.

### 2.11 Data analysis

The statistical analysis and graphical representation of data were performed using Prism version 10.0.2 (GraphPad Software, San Diego, CA, USA). A reverse Kaplan–Meier time-to-event was used to present differences in time-to-islet engraftment between diabetic NHP recipients. All histopathological scoring was performed by a board-certified veterinary pathologist with graft assessment including viability, islet fragmentation, insulin production, and inflammatory infiltration of cell product.

## 3 Results

### 3.1 Targeted mapping technique of left lateral liver lobe

**Figure 2:**
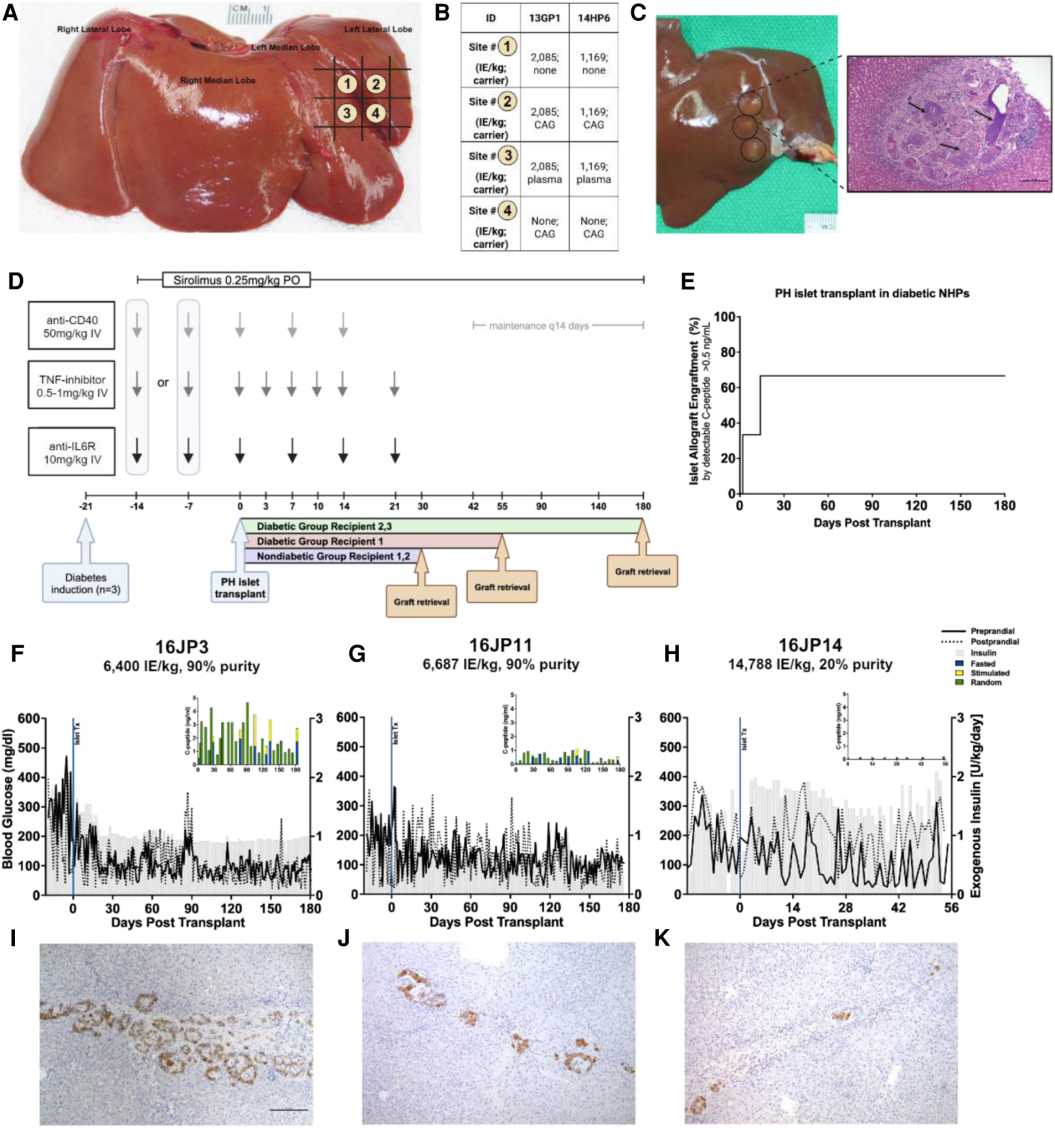
Overview of PH surface islet transplantation and targeted mapping technique. (**A**) Representative schematic of targeted mapping technique using the left lateral liver lobe. Numbers correspond to different map site constructs spatially distributed by grid. (**B**) Islet-carrier constructs for each map site for each recipient. Islet dose presented in islet equivalents per kilogram. CAG, capillary alignate hydrogel; Plasma, autologous plasma (**C**) *Left.* Liver at necropsy showing islet grafts on PH surface. Grafts are circled in black. *Right.* H&E staining of representative islet-capillary alginate hydrogel map site, black arrows point to areas of aggregated hydrogel. (**D**) Study design overview for NHP recipients including immunosuppression protocol and time of graft retrieval. (**E**) Reverse Kaplan–Meier estimate of time to islet engraftment in NHPs calculated from the date of transplantation to the date of engraftment as measured by C-peptide ≥0.5 ng/ml. (**F**) Daily measures of preprandial (solid line) and postprandial glucose (dashed line) in mg/dl and exogenous insulin requirements (gray) in U/kg with inset showing human C-peptide (ng/ml) measured randomly (green), under fasting (blue), or stimulated (yellow) conditions by days post-transplant in recipient 16JP3 with a dose of 6,400 IE/kg, (**G**) in recipient 16JP11 with a dose of 6,687 IE/kg, and (**H**) in recipient 16JP14 with a dose of 14,788 IE/kg, low purity. (**I**) Insulin immunohistochemistry staining for islets in recipients 16JP3, (**J**) 16JP11, and (**K**) 16JP14.

The targeted mapping technique was applied in two non-diabetic NHPs with the intent to functionalize the PH surface to improve islet survival while testing multiple carriers and conditions in a single animal, thereby reducing the overall number of NHPs needed by maximizing conditions that can be studied exposed to the same immune response for direct comparisons. An anatomic map of the left lateral lobe (66) is created depicting the spatial orientation for each islet-carrier construct facilitating graft retrieval later (Figure 2A). Using a small upper-midline laparotomy, the left lateral liver lobe is extracorporeally delivered and the islet-carrier constructs are injected under the liver capsule, forming discrete wheals spaced approximately 5 mm apart in a planned grid pattern. Each wheal contains a different experimental condition including islets and carrier (isolation media, autologous plasma, capillary alginate hydrogel) either together or alone (Figure 2B) and these wheals are identifiable at the time of retrieval (Figure 2C). Islet purity was 95% for both recipients and the total islet dose was equally divided across conditions.

### 3.2 Biocompatibility and safety of the PH surface for islet transplantation

Biocompatibility and safety were assessed after 30 days in immunosuppressed non-diabetic NHPs (Figure 2D). The procedure was well tolerated, no adverse events associated with the transplantation were experienced, and the NHPS’ weight remained stable throughout the 30 days (Figures 1B,C).

**Figure 3:**
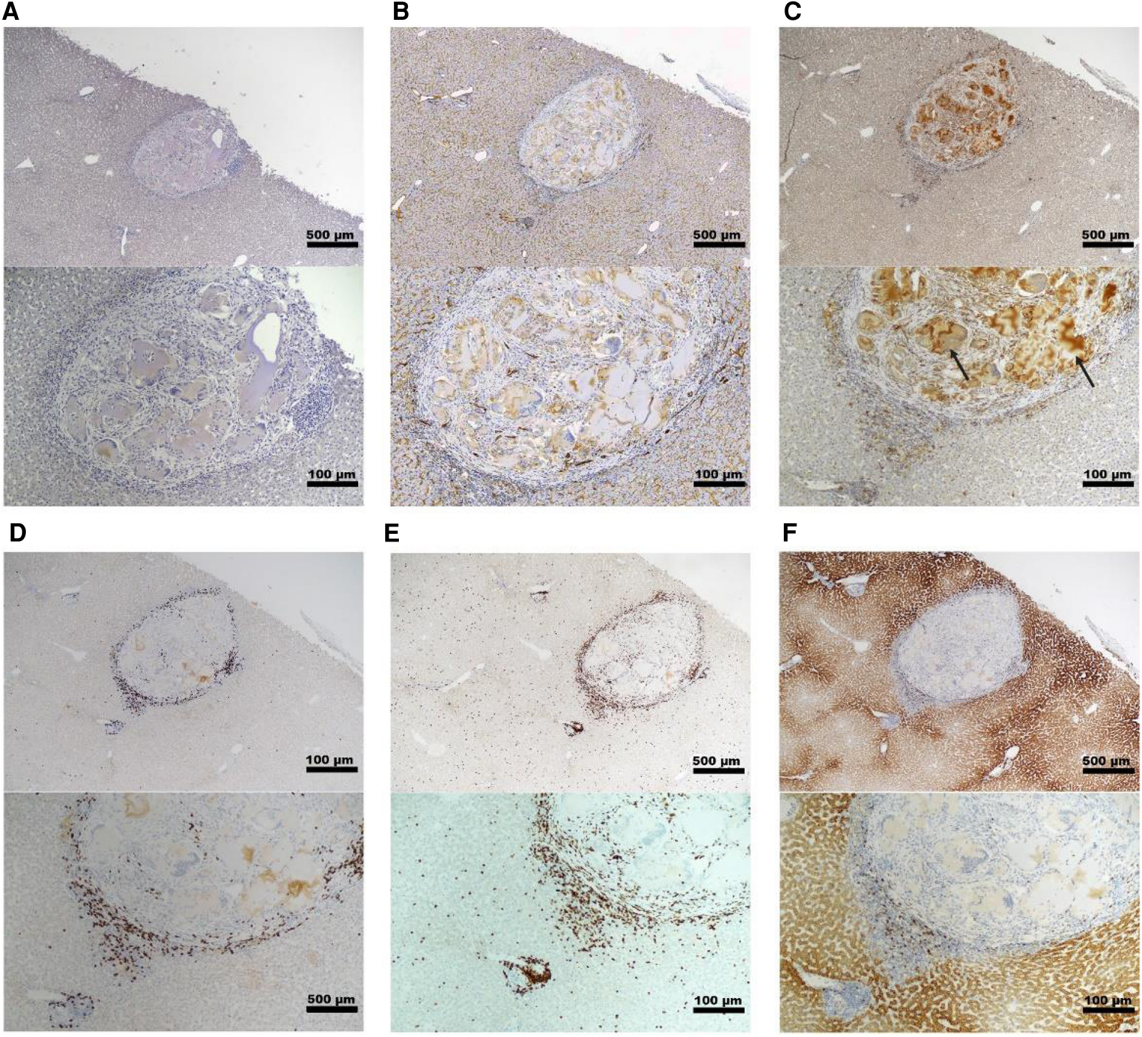
Representative histology of graft site in a non-diabetic, targeted mapping NHP recipient. Sections taken from the site injected with islet-capillary alginate hydrogel construct at 4× (*top*) and 10× (*bottom*) magnification with various stains including (**A**) insulin staining, (**B**) CD31 IHC staining for endothelial lined blood vessels, (**C**) Iba-1 IHC staining for macrophages, with black arrows pointing to the areas of background staining by capillary alginate hydrogel, (**D**) CD3 IHC staining for T-cells, (**E**) CD20 IHC, and (**F**) CD79a staining for B-cells. Scale bar: 500 µm at 4× magnification; 100 µm at 10× magnification.

Histopathology and immunohistochemistry were performed on the PH surface after the graft retrieval at 30 days. A histopathologic evaluation demonstrated a thin layer of fibrosis surrounding the graft site, with mild to moderate macrophages and a few lymphocytes present in the islet-only and islet-autologous plasma constructs. In the conditions utilizing capillary alginate hydrogel (Figure 1A), the hydrogel was evident as homogenous material within the graft site extending into the hepatic parenchyma surrounded by multinucleated inflammatory giant cells (Figure 2B). Around this, a zone of fibrosis was seen with a moderate amount of macrophages and lymphocytes as well as a few polymorphonuclear leukocytes and eosinophils (Figures 3C–F). CD31 immunohistochemistry identified endothelial lined vessels and demonstrated prominent microvascularity at the graft sites (Figure 3B). Overall, the PH surface demonstrated significant vascularization at the graft sites with some inflammatory cells present in the conditions without hydrogel whereas a more robust immune response was seen in the hydrogel constructs as evidenced by the number and diversity of inflammatory cells at the site. Interestingly, there were no identifiable islet cells on histology (Figure 3A) across conditions, despite adequate immunosuppression.

### 3.3 Graft survival and function after PH surface islet transplantation in diabetic NHPs

Following feasibility testing with targeted mapping in non-diabetic NHPs, three streptozotocin-induced diabetic NHPs were chosen to undergo PH surface islet transplantation to evaluate long-term graft survival and function. These were similarly immunosuppressed using an anti-inflammatory induction regimen with rapamycin maintenance and co-stimulatory blockade for both induction and maintenance therapy (Figure 2D). Islets with an autologous plasma carrier were used for the PH surface transplantation based on the histological evaluation during targeted mapping demonstrating a greater inflammatory response and fibrosis with the use of hydrogel. Furthermore, autologous plasma proved easy to handle and inject with more control over spatial distribution in comparison to naked islets in media. The dosage and purity of islets transplanted can be found in Table 1.

**Table 1:** Diabetic cynomolgus macaque demographics and transplant characterization.

ID	Sex	Age (year)	Weight (kg)	Islet purity (%)	Islet dose (IE/kg)	Graft survival (days)	Graft retrieval (POD)
16JP3	Female	4.8	3.94	90	6,400	>183	183
16JP11	Female	4.8	4.11	90	6,687	>176	176
16JP14	Female	4.6	3.36	20	14,788	0	55

IE/kg, islet equivalents per kilogram; POD, post-operative day.

**Figure 4:**
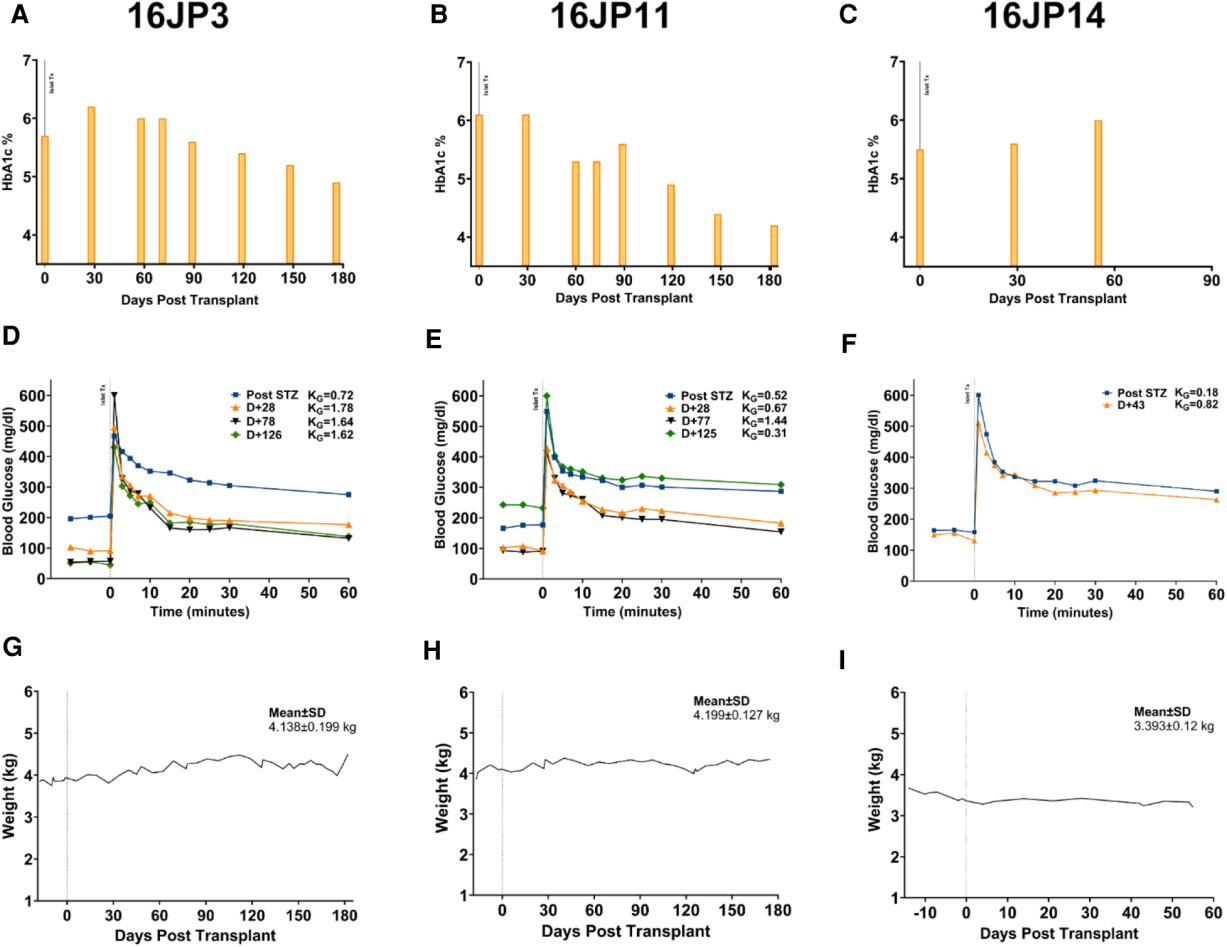
Metabolic effects of PH surface islet transplant in diabetic, immunosuppressed NHP recipients. (**A**) HbA1c % measured at transplant and up until graft retrieval for 16JP3, (**B**) 16JP11, and (**C**) 16JP14. (**D**) IVGTT measurements taken after STZ (blue), 28 (orange), 78 (black), and 126 (green) days after transplant for 16JP3, (**E**) after STZ (blue), 28 (orange), 77 (black), and 125 (green) days after transplant for 16JP11, (**F**) after STZ (blue) and 43 (orange) days after transplant for 16JP14. KG, glucose disappearance rate (%/min). Daily weights for (**G**) 16JP3, (**H**) 16JP11, and (**I**) 16JP14. Mean weight and standard deviation presented within the graph.

Two of the three recipients (16JP3 and 16JP11) demonstrated islet engraftment within 14 days (Figure 2E) and long-term graft survival as measured by C-peptide (>0.5 ng/ml) through 180 days (Figures 2F,G). Both 16JP3 and 16JP11 demonstrated improved trends in median preprandial glucose compared to pretransplant (98.0 vs. 300.5 mg/dl; 111.5 vs. 194.5 mg/dl, respectively) and in median postprandial blood glucose compared to pretransplant (91.0 vs. 194.0 mg/dl; 123.5 vs. 130.0 mg/dl, respectively). 16JP3 and 16JP11 also demonstrated decreasing daily exogenous insulin requirements compared to pretransplant (0.94 vs. 1.425 U/kg; 0.59 vs. 0.71 U/kg, respectively) (Figures 2F,G). 16JP3 had a 31% reduction in HbA1c after 180 days while 16JP11 had an overall 14% reduction in HbA1c after 180 days (Figures 4A,B). Improved glucose disposal was seen through day 126 for 16JP3 and day 77 for 16JP11 as measured using IVGTT compared to pretransplant (Figures 4D,E).

One recipient (16JP14) received a low purity islet product (20%) and did not have meaningful function throughout the post-transplant period; therefore, the evaluation and testing were only carried out through day 55. Indeed, C-peptide was <0.5 ng/ml through the entire post-transplant period (Figure 2H). No meaningful improvements in pre- or postprandial glucose, exogenous insulin requirements, glucose disposal, or HbA1c were demonstrated either (Figures 2H, 4C,F,I).

**Figure 5:**
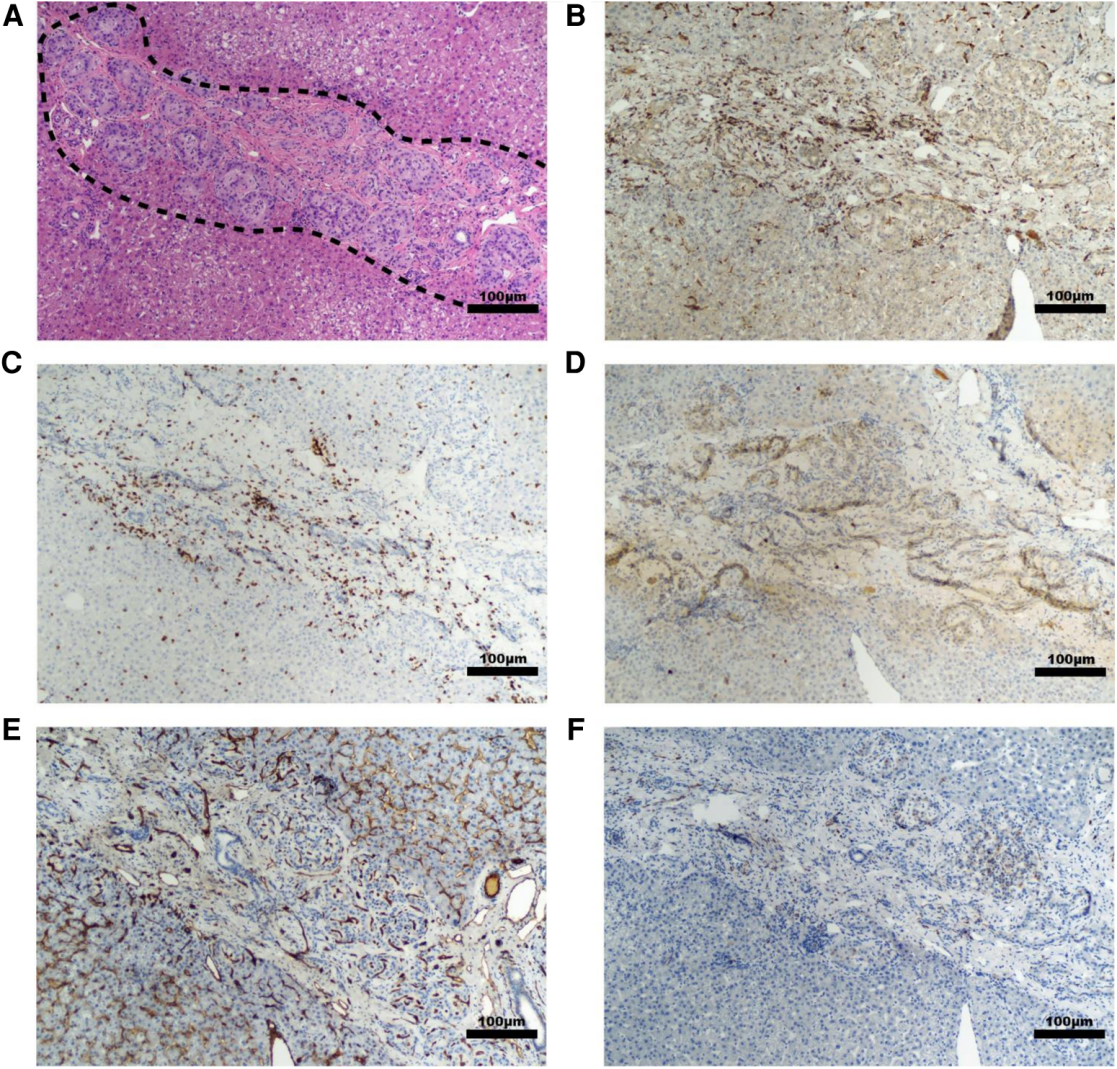
Representative histology of graft site in a diabetic NHP recipient. Sections taken from the site injected with islet-autologous plasma at 10× magnification with various stains including (**A**) H&E, (**B**) Iba-1 IHC staining for macrophages, (**C**) CD3 IHC staining for T-cells, (**D**) CD20 IHC staining for B-cells, (**E**) CD31 IHC staining for endothelial lined blood vessels, and (**F**) β3 tubulin IHC staining for neurons. The dashed line highlights the cluster of islets. Scale bar 100 µm.

Histopathological findings in diabetic recipients revealed intact, engrafted islets that were organized in loose clusters (Figure 5A). Interestingly, despite perihepatic, subcapsular injection, many islet clusters were located deeper around portal tracts and zones. In all diabetic recipients, histology revealed mild fibrosis with a few inflammatory cells present and little evidence of immune rejection around the islet grafts (Figures 5B–D). Moderately to strongly positive insulin staining was seen in recipients 16JP3 (Figure 2I) and 16JP11 (Figure 2J). Conversely, while recipient 16JP14 showed relatively intact islets, there were overall smaller numbers and weaker positive insulin staining compared to the other recipients (Figure 2K). Prominent microvascularity was detected in the graft sites for all recipients and there was evidence of innervation within the islets or in the surrounding tissues (Figures 5E,F).

## 4 Discussion

The purpose of this study was to evaluate the capability of the PH surface to support transplanted islet cells in the translationally relevant NHP model. While intraportal injection remains the gold standard for islet transplantation, issues related to significant immediate graft loss, relative hypoxia, IBMIR as well as portal vein thrombosis and hypertension have led investigators to seek other potential sites for islet transplant. While some of these extraportal sites have demonstrated some advantages over the traditional transplant site, at this time, none have been shown consistent superiority to portal vein delivery. These reasons led our studies to investigate the PH surface as a potential extraportal site. Our results indicate that the PH surface is able to support islet cell survival through 180 days with detectable improvements in metabolic parameters using conventional, commercially available immunosuppression.

We chose the PH liver surface for several reasons but primarily because of the superior vascularization of the liver, an issue for many of the previously studied extraportal sites (28–30, 39, 67–69). The PH surface places the graft adjacent to the liver parenchyma, which has a dual blood supply, receiving arterial blood from the hepatic artery and deoxygenated blood from the portal vein. This utilizes an intrinsic vascular bed, avoiding the need to prevascularize the space before transplant, such as for subcutaneous sites (67, 68, 70). Studies evaluating liver parenchymal oxygen tension have shown pO2 in the range of 42–57 mmHg (44, 71, 72), which is similar to, if not slightly better than, portal vein oxygen partial pressure. This supports islet survival during revascularization while avoiding direct contact with blood, protecting the graft from IBMIR. As insulin is normally secreted in a pulsatile manner from the pancreas into the portal vein and then to the liver, the PH site allows for physiologic insulin secretion given the proximity to the portal drainage and reinnervation of the site could recapitulate the pulsatility of secretion.

In addition to the oxygenation and vascularization advantages, the PH liver surface allows for easier access to the islet graft. In our study, we were able to access the left lateral liver lobe (Figure 2A) through a small upper midline incision of approximately 6 cm in comparison to a large laparotomy or bilateral subcostal incision as seen in total pancreatectomy with islet auto transplantation (TPIAT) (73). The PH site also lends itself to percutaneous ultrasound-guided access, allowing for a potential percutaneous PH surface islet injection as in the case of a clinical allogeneic islet portal transplant (19). This ease of access simplifies transplantation but also allows for graft biopsy or retrieval, which is not possible in an intraportal islet transplant. Furthermore, if a graft retrieval or biopsy requires a more extensive liver resection, this can be done without significant detriment to the liver, which requires only a 20% functional liver remnant in order to regenerate (47, 74).

In our study, we first attempted to functionalize and improve the conditions of the PH liver surface for islet cells. Native islet survival, in part, relies on the ECM to create a particular spatial distribution in the pancreas, allowing for autocrine and paracrine signaling with neighboring cells (75); isolation of the cells and a loss of ECM leads to a form of cell death (76, 77). As the isolation process removes much of the ECM and structure, we hypothesized that the use of a capillary alginate hydrogel may function as a scaffold for the islets and improve their survival and functionality. Some studies have also demonstrated increased growth factor release, wound healing, and vascularization using autologous plasma positioned this as an important carrier to study (78–80). Capillary alginate hydrogels were chosen as a potential carrier as they have been investigated in a wide array of biomedical contexts, including the 3D culture of embryonic stem cells (57), as an injectable neural stem cell delivery and scaffolding system (60), as an injectable post-myocardial infarction therapeutic (63), as a treatment for full-thickness skin wounds (62), to engineering *in vitro* functional 3D nerve tissue models (57), as new bioinks for 3D printing (61), and, recently, to engineering human tissues for direct arthropod biting and blood feeding (62). As inter-donor and recipient variability may confound results, particularly in the small group sizes characteristic in pilot NHP studies, we utilized the targeted mapping technique allowing us to simultaneously test multiple islet-carrier constructs in the same recipient with islets from the same donor.

We evaluated the hydrogel, plasma, and naked islet constructs via targeted mapping technique in two non-diabetic NHPs with planned graft retrieval at day 30. The naked islet and autologous plasma constructs showed minimal fibrosis and immune cell infiltration whereas the hydrogel constructs demonstrated greater and more diverse inflammatory cell infiltration at the site and around the hydrogel with more significant fibrosis. This finding is interesting given that multiple studies have demonstrated the utility of alginate hydrogel for encapsulation and subsequent implantation of islets in various models (44, 64, 81). In studies using alginate hydrogel in the kidney subcapsular space, these use alginate hydrogel as a means for microencapsulation of islets (81–84), whereas in this study, islets and hydrogel were mixed before injection under the liver capsule. In contrast to microencapsulated islets, the histology shows large, homogenous areas of aggregated hydrogel; these areas are surrounded by zones of fibrosis and inflammation (Figure 2C). Though the mechanism by which hydrogel stimulates this inflammatory response is unclear, it has a similar histologic appearance of a foreign body reaction and perhaps the large, accumulated areas of hydrogel in the PH space are being treated as such. Other studies have demonstrated how alginate hydrogel stimulates an inflammatory response leading to fibrosis and islet death (44, 85). Though spatially separated by a few millimeters, we hypothesize that the capillary alginate hydrogel may have a systemic adjuvant effect on the immune system and in combination with relatively low islet doses per injection site (1,169–2,085 IE/kg), likely explained the lack of islets seen across conditions.

Building on these results, we wanted to assess the impact of the PH liver surface on long-term survival and the metabolic effects in diabetic recipients using the best condition, autologous plasma as a carrier for the islets. In a similar procedure to the targeted mapping studies, three diabetic, immunosuppressed NHPs underwent PH transplant with allogenic islet-plasma single-site injection with planned graft retrieval at day 180. While no recipients achieved insulin independence, two recipients (16JP11 and 16JP3) had positive C-peptide levels at day 180 with improved HbA1c at the time of graft retrieval compared to the day of transplant (Figures 2F,G, 4A,B). 16JP3 demonstrated islet engraftment soon after the transplant while 16JP11 demonstrated engraftment at day 14 (Figure 2E). Both also had improved glucose disposal (Figures 4D,E) though 16JP11 eventually had loss of graft function despite graft survival through day 180. On histology, loose clusters of intact islets were seen with moderate to strongly positive insulin staining observed (Figures 2F,J, 5A). Overall, there was minimal fibrosis or inflammation detected with minimal evidence of rejection. IHC staining also showed microvascular formation and evidence for innervation of the sites (Figures 5E,F).

Conversely, recipient 16JP14 did not demonstrate significant C-peptide levels and was unable to achieve a metabolic benefit after transplant (Figure 2H); graft retrieval occurred on day 55 as a result. Interestingly, 16JP14 had the highest islet dose of the three recipients (14,788 IE/kg); however, islet purity was only 20% compared to the 90% purity of the other two recipients. Similar to the other recipients with diabetes, IHC showed vascularization and innervation at the site with evidence of intact islets. However, in comparison, there were relatively few islets seen with minimal positive insulin staining (Figure 2K). We suspect that the significantly poor purity of the islets resulted in the overall lack of function and benefit after transplant.

In all three recipients with diabetes, there was evidence of the graft extending deeper into the parenchyma despite injection under the liver capsule; the reason for this is not entirely clear. At the time of transplant, it could be that the initial puncture of the liver capsule was deeper into the parenchyma and could have created a tract for islets to migrate after injection. Regardless, it is likely of little clinical consequence in terms of graft survival and function or in terms of safety as there were no adverse events related to this. Indeed, in a mouse model, one group has demonstrated the efficacy of islet transplant within a hepatic sinus tract (HST) created in the liver parenchyma (86, 87).

As the portal vein is used in both allogenic islet transplants (17) and autologous islet transplants, such as in TPIAT (88), the surgeon must continuously monitor and account for changes in portal venous pressure (PVP). A rise in PVP is a known consequence of islet transplantation, with the potential for portal hypertension, bleeding complications, and portal vein thrombosis (39, 89–92). Impurities in islet preparations are a known risk factor for increased PVP (93), a particular concern in TPIAT where islet cell yield is typically low due to chronic pancreatitis and thus, purification is often not performed to maximize the islet dose (93, 94). As a result, this rise in PVP often limits the amount of islet product able to be infused, with the remaining preparation typically dispersed freely into the intra-abdominal cavity (95, 96), an inferior site from a functional and histological standpoint compared to others (30, 97).

In this regard, the PH surface may represent a viable site to maximize the functional result from an autologous islet transplant in particular, compared to free dispersal into the peritoneal cavity, though the site is not capable of “rescuing” highly impure products, as seen in recipient 16JP14. Previously, the kidney subcapsular space was considered to be a potential site for islet transplantation but was unsuccessful in NHPs even using doses that were approximately twofold higher than those routinely successful in intraportal transplant (69). Similarly, in humans, the renal subcapsular site was inferior to intraportal transplant and resulted in only marginal C-peptide secretion with no appreciable metabolic benefit (98, 99). Both the surgical invasiveness necessary to expose the kidney and the prevalence of diabetic nephropathy in potential recipients continues to limit the feasibility of this site (12, 30). In contrast, the PH surface advantages the dense vascular network of the dual blood-supplied liver while the close proximity to the portal system preserves physiologic insulin kinetics, as demonstrated in the response to glucose challenge, given the islet dose is optimized. Furthermore, as there is a direct injection into the portal system when the PH surface is being utilized, there is no increase or change in PVP. During TPIAT, the PH surface is readily accessible whereas the retroperitoneum must be entered to access the kidney. For these reasons, as well as the demonstrated long-term function, survival, and vascularization of the PH-transplanted islet grafts, the PH may be an easy and advantageous site for the transplantation of the remaining islet preparation when the PVP prohibits further portal vein infusion during TPIAT. By harnessing all available islet products for transplantation, this could not only improve the metabolic benefits gained from transplant, but also increase the possibility of insulin independence.

### 4.1 Limitations

Given the exploratory nature of this study with the use of NHPs, the number of subjects was relatively small. While we were able to test new techniques and various conditions, the adjuvant effect that may present with certain hydrogels limits the evaluation of immune response to the immediate local reaction in the targeted mapping technique. Inflammation and rejection were only evaluated through histology at the time of graft retrieval; therefore, the immune response throughout the study period or soon after transplant is unclear. In the future, serologic markers of inflammation and immunoactivity in combination with potential serial graft biopsies would help shed light on the dynamic immunologic landscape after PH transplants.

## 5 Conclusions

We demonstrate the ability of the perihepatic liver surface to support the long-term function and survival of transplanted allogenic islet cells in NHPs on a conventional, clinically relevant immunosuppression regimen. Initial targeted mapping studies allowed for the simultaneous testing of multiple conditions to rule out islet-carrier constructs for additional testing in the more stringent STZ-induced NHP model and demonstrated the safety of PH surface islet transplantations. In diabetic recipients receiving standard purity islet products, PH islet transplants demonstrated islet survival through the day 180 endpoint, with improvements seen in blood glucose, exogenous insulin needs, HbA1c, and glucose disposal. Unlike intraportal islet transplants, the PH surface is accessible, allowing for graft biopsy or retrieval. While further work is still necessary, the PH surface may be a clinically relevant site for transplanting remaining islets after the portal venous pressure limit is reached during traditional portal islet transplants.

## Data Availability

The raw data supporting the conclusions of this article will be made available by the authors, without undue reservation.
